# Normocalcemic parathyroid carcinoma: an unusual clinical presentation

**DOI:** 10.1186/1477-7819-4-10

**Published:** 2006-02-21

**Authors:** Corrie L Messerer, Samuel P Bugis, Chris Baliski, Sam M Wiseman

**Affiliations:** 1 Faculty of Medicine, University of British Columbia, Vancouver, BC, Canada; 2 Dept of Surgery, St. Paul's Hospital, University of British Columbia, Vancouver, BC, Canada

## Abstract

**Background:**

Parathyroid carcinoma is a rare cause of primary hyperparathyroidism and may be associated with significant disease related morbidity and mortality. Preoperative diagnosis remains a challenge, which may jeopardize appropriate and successful patient treatment.

**Case presentation:**

We report a case of parathyroid carcinoma diagnosed in a 60-year-old woman that presented with a tender nodule located at the left lower thyroid pole and had been present for several years. Ultrasound examination revealed a 2.7 × 1.6 × 2.7 cm mass within the lower left lobe of the thyroid with cystic and solid areas. Lab measurement of the intact PTH level revealed it to be three times the upper limit of normal and the serum calcium level was within normal limits. A left thyroid lobectomy and isthmusectomy was carried out. Histopathological evaluation was diagnostic for a parathyroid carcinoma. At greater than two years of follow-up, the patient has had no evidence of disease recurrence and her serum PTH and calcium levels have remained within normal.

**Conclusion:**

Parathyroid carcinoma is a rare endocrine tumor which must be considered in the differential diagnosis of a nodular thyroid mass. *En bloc *resection remains the treatment of choice for this malignancy. Disease prognosis is influenced by the extent of the initial resection, the presence of metastases, and adequate long-term follow-up.

## Background

Parathyroid carcinoma (PC) is responsible for fewer than 1% of cases of primary hyperparathyroidism [1]. The majority of patients with PC present in their fifties, and unlike parathyroid adenoma or hyperplasia which predominantly effect women, PC appears to be equally distributed between the genders [1]. Patients may present with one or more characteristic features of PC, which include severe symptoms of primary hyperparathyroidism and significantly elevated serum PTH (Table 1). Parathyroid carcinoma may arise as a sporadic disease, with familial hyperparathyroidism [2], with hyperparathyroidism-jaw tumour syndrome [3], or with multiple endocrine neoplasia type 1 (MEN-1) [4].

**Table 1:** Clinical distinctions between primary hyperthyroidism and parathyroid carcinoma. Adapted from [38].

	**Parathyroid carcinoma**	**Primary hyperparathyroidism**
Palpable neck mass	50%	<10%
Hypercalcemia (normal = 8.5–9.9 mg/dL)	> 14 mg/dL	11–12 mg/dL
↑ PTH (normal = 10–65 pg/mL)	3–10× normal	2× normal
Renal impairment (nephrilithiasis, nephrocalcinosis, impaired GFR)	32–84%	<20%
Skeletal abnormalities (osteitis fibrosa cystica, diffuse spinal osteopenia, subperiosteal bone resorption, salt-and-pepper skull)	44–91%	<10%

In this article we report on a case of PC that presented as a tender thyroid mass. It was resected and diagnosed postoperatively as PC. The patient has remained disease free for greater than two years postoperatively.

## Case presentation

A 60-year-old woman was referred for evaluation of a tender nodule in the left lobe of the thyroid. She had no prior history of head and neck radiation and no family history of thyroid or parathyroid disease. The patient had noted little change in lesion size but persistent pain, aggravated by talking, which was troublesome for her. She did not have any complaints of voice change. She had initially been investigated 5 years earlier with complaints of pain radiating over the left side of her face. At that time a small firm nodule in the left thyroid lobe was discovered and a needle biopsy found no evidence of malignancy. At that time it was diagnosed as a benign nodular goiter and observation was recommended.

An ultrasound identified a left lower thyroid lobe mass measuring 2.7 × 1.6 × 2.7 cm with cystic and solid components. Her serum ionized calcium level was normal and her intact PTH was elevated to three times the upper limit of normal. Due to ongoing facial pain a left thyroid lobectomy and isthmusectomy was carried out. Intraoperatively, a firm multinodular left thyroid lobe was appreciated. There were no abnormal central compartment lymph nodes identified or removed at surgery.

**Figure 1:**
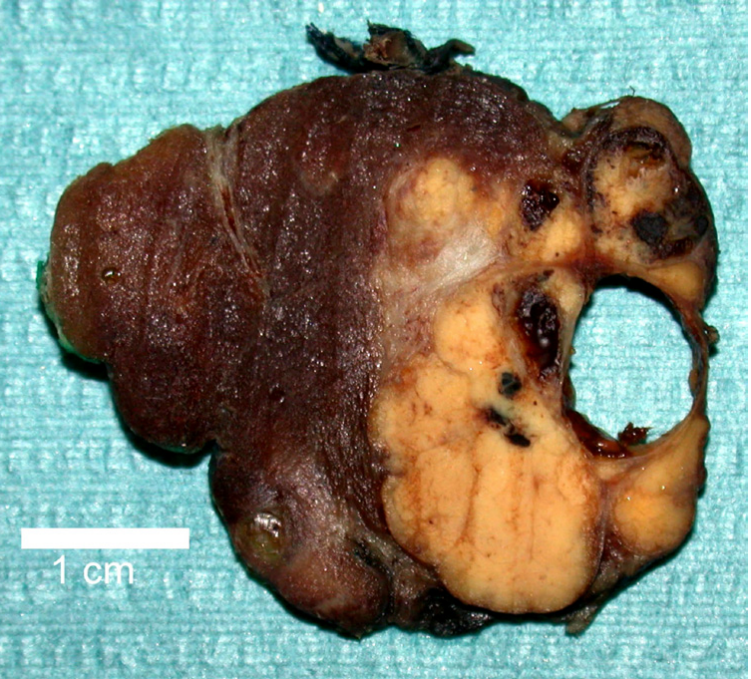
Gross appearance of parathyroid tumour and thyroid lobe.

**Figure 2:**
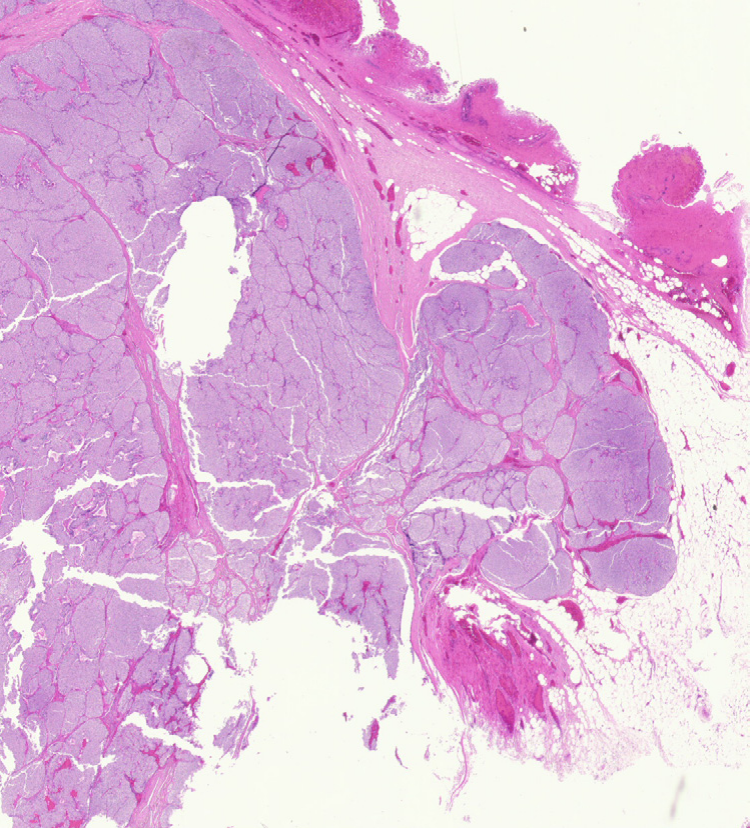
Parathyroid carcinoma show mushrooming invasion through capsule into the surrounding fat. (H&E ×40).

Pathological examination of the specimen revealed a large, yellowish tumor measuring 2.7 × 2.0 × 2.0 cm that was pushing into the left thyroid lobe, but separated from it by a fibrous capsule (Figure 1). The mass was noted to be composed of monomorphous parathyroid cells without significant necrosis or mitotic activity. There was invasion into the thyroid capsule and into adjacent fatty tissue. No vascular invasion was noted (Figure 2). A cystic goiter was also noted in the left thyroid lobe. Further immunohistochemical studies found the tumour did not express: Thyroid Transcription Factor – 1, Calcitonin, and carcinoembryonic antigen (CEA), but did express Chromagranin, Synaptophysin, Bcl -2 and Cytokeratin 7.

Postoperatively the patient's parathyroid hormone normalized and her ionized serum calcium level remained within normal limits. Her postoperative course was uneventful and she has remained disease free greater than 27 months postoperatively.

## Discussion

PC is a rare cause of primary hyperparathyroidism. Its rarity makes preoperative diagnosis difficult and much of our current knowledge of this cancer is derived from retrospective case studies. In the vast majority of patients, PC tends to be a functional malignancy. Non-functional parathyroid cancers are especially uncommon, accounting for approximately 1.9% of parathyroid tumors and represent a [1] prognostic indicator of poor outcome. Patients with non-functional tumors tend to present later in their disease course, and it is speculated that these cancers may be more aggressive. At presentation approximately 40% of PC patients will have profound hypercalcemia and a palpable neck mass [1]. PC rarely presents with cervical, mediastinal or distant metastatisis. Koea *et al*, [1] reported that of 301 PC patients, 10 patients (2%) had regional nodal metastasis at initial presentation and 9 [2] patients (2%) were diagnosed with distant metastasis to lung or bone at their initial presentation.

Interestingly, several reports have noted that the lower parathyroid glands appear to have an increased incidence of PC [1,5-7]. The lower parathyroid glands are derived from the third branchial arch, a different embryologic origin then the superior thyroid glands, which are derived from fourth branchial arch. It is unknown why the inferior parathyroid glands appear to have an increased predisposition for carcinoma development.

While the etiology of PC remains poorly defined, recent studies of its molecular pathogenesis have implicated several genes. These genes include: cyclin D1, [8], p53 [9] and the retinoblastoma tumour suppressor gene [10]. Moreover, individuals with familial hyperparathyroidism, MEN- 2 [4], and hyperparathyroidism-jaw tumour syndrome [3] appear to have an increased predisposition for the development of PC.

Intraoperatively PC presents as a hard, lobulated mass that invades the thyroid [1,11-13]. The neoplasm frequently is found to adhere to surrounding structures including the thyroid gland, the strap muscles, the recurrent laryngeal nerve, esophagus, and trachea [1,11-13]. The diagnosis of parathyroid carcinoma is based on the histopathological criteria established by Shantz and Castleman in 1973 [14]. These histopathologic criteria include: the presence of a fibrous capsule or fibrous trabeculae, trabecular or rosette-like cellular architecture, the presence of mitotic figures, and the presence of capsular or vascular invasion. These criteria have been challenged by McKeown *et al *[15], who noted that it is critical to differentiate between mitotic activity in tumor parenchymal cells versus endothelial cells. They further suggested that cellular pleomorphism and atypia are unreliable indicators of malignancy in this endocrine cancer. In 1993 Bondeson and colleagues [16] addressed this controversy by careful analysis of 56 histopathological specimens of metastatic PC. They found that mitotic activity in these carcinomas was highly variable with half of cases had mitotic rates comparable to benign parathyroid lesions. Further, while fibrosis, usually with the capsule, was observed in 80% of these carcinomas, the authors reported that a trabecular architecture pattern was not diagnostic of PC, as it was also observed in parathyroid adenomas and hyperplasia.

Recent immunohistochemical studies have attempted to distinguish benign from malignant parathyroid neoplasms by utilizing immunohistochemical markers. One study [17] revealed that 76% of the patients with typical parathyroid adenoma had a phenotype of p27(+)bcl-2(-)Ki-67(-)mdm2(+) compared to none of those patients diagnosed with PC. Other studies have found that more that half of patients diagnosed with PC showed one of three molecular phenotypes: p27(+)bcl-2(-)Ki-67(+)mdm2(-) (9%), p27(-)bcl-2(-) Ki-67(-)mdm2(-) (27%), and p27(-)bcl-2(+)Ki-67(+)mdm2(-) (18%). None of these phenotypes were observed in patients diagnosed with parathyroid adenomas. These studies demonstrated the potential clinical utility of immunohistochemical markers in differentiating benign versus malignant parathyroid disease. However, it is still generally agreed that that invasion into surrounding tissues, the presence of a fibrotic capsule, and nuclear atypia remain the most accurate predictors of malignant histology in parathyroid neoplasms [1].

Currently the gold standard for the treatment of parathyroid carcinoma is *en bloc *surgical resection. Surgery may require thyroid lobectomy, isthmusectomy and possibly central neck nodal dissection. Removal of all tissue to which tumour is adherent, including the recurrent laryngeal nerve, trachea, or oesophagus, may be necessary [1,7,18,19]. PC patients have the highest chance of cure if *en block *resection is carried out at their initial operation. Controversy exists as to whether the patient in whom malignancy is recognized after tumorectomy requires reoperation with resection of all structures adjacent to where the tumour was originally located. Some investigators have suggested that in these cases en bloc resection may be postponed until tumour recurrence is recognized by rising calcium levels [20].

Intraoperative PTH (IPTH) measurement is an emerging technique for optimizing tumour removal while minimizing the invasiveness of parathyroid surgery. Multiple studies have investigated its use in the surgical management of primary hyperparathyroidism but its role in the management of PC remains unclear. Current literature, however, suggests that with conservative PC resection is associated with a significant risk of capsule rupture and subsequent local dissemination of the tumour [20]. There have also been several reports of false-positive phenomena in cases of multiple gland pathology including double adenomas, and combination adenoma and hyperplasia, wherein the PTH level decreases to within the normal range, but begins to increase post-operatively [21,22]. It can be postulated that IPTH measurement may be of limited application to PC when metastases are present.

Historically, radiotherapy and/or chemotherapy have had little role in the treatment of PC and have generally been utilized for palliation of patients who are not candidates for surgery [5,14,23-25]. Several recent studies, have suggested that adjuvant radiotherapy may lengthen the disease free survival period in PC patients [26-30].

Chemotherapy has had a limited role in the treatment of PC. Several regimens (nitrogen mustard; vincristine, cyclophosphamide and actinomycin D; adriamycin, cyclophosphamide, and 5-fluorouracil; and adriamycin alone) have been unsuccessful [23,31,32]. Partial responses to dacarbazine, both alone and in combination with other drugs, have been reported [33-36]. Several case reports have documented rare responses to other chemotherapy regimes [25,37].

Disease recurrence of PC is common and reported rates range from 33% to 78% [6,12,25,38,39]. It has been suggested that the variability in these estimates are due to the application of different diagnostic criteria in multiple small retrospective studies [18]. Elevated serum PTH and calcium levels are present in persistent or recurrent disease in greater than half of patients with parathyroid carcinoma who are not cured by their initial treatment [12]. Most PC recurrences occur 2 to 3 years after surgery [39], although recurrence has been reported up to 23 years after an initial surgical resection [12]. It is also important to recognize that patients may have non-functioning metastatic PC. Approximately 25% of PC patients develop distant metastasis during follow-up, most commonly to the lungs and bones, and less frequently to the liver [12,13]. Data from a United States National Cancer Database report suggests the five and ten year survival rates for parathyroid carcinoma are 85% and 49%, respectively [40].

Patients with parathyroid carcinoma more commonly die from refractory hypercalcemia rather than the tumour burden itself [25, 41]. Therefore an important aspect of medical management of these patients relates to the treatment of hypercalcemia. This includes potentiation of calciuresis with saline infusion and loop diuretics [42], as well as application of agents that interfere with osteoclast-mediated bone reabsorption. These include several groups of drugs that include: bisphosphonates, plicamycin (mithramycin), calcitonin and calcimimetics [25, 41, 42, 43, 44].

## Conclusion

PC is a rare ensdocrine cancer with an insidious clinical course that is often misdiagnosed as primary hyperparathyroidism secondary to parathyroid adenoma or hyperplasia. Most commonly PC patients present with a neck mass and signs of severe hypercalcemia with metabolic bone disease and renal failure. As was the case for our patient, normocalcemia at presentation is unusual. Incomplete tumor resection leads to recurrence and mortality rates approaching 50%. Thus, preoperatively differentiating between benign and malignant parathyroid neoplasms, though a challenge, is important, and en bloc tumor resection offers these patients the best chance for cure.

## Competing interests

The author(s) declare that they have no competing interests.

## Authors' contributions

**CLM **reviewed the literature and drafted the manuscript.

**SMW **was involved in the conception of this work, literature review and manuscript preparation.

**CB **and **SB **assisted in manuscript revision and review.

All authors read and approved the final manuscript.
